# Sonic Hedgehog Expression is Associated with Lymph Node Invasion in Urothelial Bladder Cancer

**DOI:** 10.1007/s12253-018-0477-6

**Published:** 2018-10-25

**Authors:** Taoufik Nedjadi, Nada Salem, Dareen Khayyat, Ahmed Al-Sayyad, Adel Al-Ammari, Jaudah Al-Maghrabi

**Affiliations:** 1 King Abdullah International Medical Research Centre,, King Abdulaziz Medical City, Ministry of National Guard-Health Affairs, Jeddah, Kingdom of Saudi Arabia; 2 Center of Excellence in Genomic Medicine Research, King Abdulaziz University, Jeddah, Saudi Arabia; 3 King Fahd Medical Research Centre, King Abdulaziz University, Jeddah, Saudi Arabia; 4 Department of Urology, King Abdulaziz University, Jeddah, Saudi Arabia; 5 Department of Urology, King Faisal Specialist Hospital & Research Center, Jeddah, Saudi Arabia; 6 Department of Pathology, King Abdulaziz University, Jeddah, Saudi Arabia

**Keywords:** Bladder cancer, Sonic hedgehog, Expression, Lymph node, Prognosis

## Abstract

Bladder cancer (BC) is a deadly disease characterized by high recurrence rates and frequent progression to an aggressive phenotype. Dysregulation of various signaling pathways have been implicated in BC tumorigenesis, however, the clinical relevance of sonic hedgehog pathway (Shh) remains under investigated. The aim of the current study was to analyze the prognostic value of Shh expression in patients with bladder carcinoma. Immunohistochemical expression of Shh was performed using tissue microarray with 128 specimens from bladder cancer patients. Kaplan-meier survival was analysed and correlation between Shh protein expression and patients’ clinicopathological parameters wasexamined using Fisher’s exact test. The immuno-staining results revealed that Shh protein exhibits cytoplasmic localization and is expressed in 49% of the analyzed bladder cancer cohort. Our data indicated that high Shh expression significantly correlated with increased lymph node metastasis (*p* = 0.02), however no association was reported between Shh expression and other clinicopatholigical parameters. High expression of sonic hedgehog was associated with lymph node invasion which may indicate that Shh might play an important role in progression and metastasis of bladder cancer.

## Introduction

According to a recent report by Antoni et al. 2017, an estimated 430,000 new bladder cancer (BC) cases have happened in the year 2012 ranking BC the ninth most common type of cancer worldwide [1]. Approximately, 75% of the newly diagnosed BC patients have non-muscle invasive tumours (NMIBC) confined to the mucosa (stages Tis, Ta and T1) and the remaining 25% of patients have muscle invasive (MIBC) or metastatic disease at initial presentation in the clinic [2]. Patients with NMIBC disease have high risk for cancer recurrence and the possibility for progression to an aggressive muscle invasive form is also high which necessitate continuous surveillance and monitoring of BC patients [3]. Kaufman et al. (2009) demonstrated that 50–70% of non-muscle invasive tumours will recur over time and approximately 10–20% will progress to muscle invasive tumours (T2 - T4) [4]. This situation necessitates an urgent need to identify a reliable prognostic biomarker that would assist in clinical setting, with the currently-used grading/ staging system, to predict for early disease development and progression [5, 6]. Thus far, several promising biomarkers have been identified and a number of FDA-approved laboratory tests have been developed for regular cancer monitoring however most of which have not been sufficiently sensitive or specific to predict clinical outcome [7–9].

Sonic Hedgehog (Shh) is a member of the hedgehog (HH) family, which also includes desert hedgehog and Indian hedgehog. Shh was first discovered as a secreted protein mediating fundamental cellular processes such as vertebrate development and embryogenesis [10]. Later, it was discovered that SHh plays also important roles in regulating cell proliferation, differentiation and cell fate [11, 12]. Recent studies have demonstrated the role of Shh in mediating the tumorigenic properties of several cancer types including lung, prostate, breast, colon, ovarian, pancreatic and hepatocellular carcinoma [13–19].

Recent data revealed that sonic hedgehog is critical regulator of several cellular processes related bladder carcinogenesis. Constitutive activation of the Shh pathway through Shh ligand binding to the trans-membrane protein Patched1 abrogates the inhibitory effect on smoothened (SMO) which undergo phosphorylation at accumulation at the cell surface. Activation of SMO generates downstream signaling cascade leading to nuclear translocation of the transcription factor Gli1 which in turn induces the transcription of several target genes including Gli1 [20–22]. A number of studies have shown that dysregulation of Shh ligand or one of its downstream mediators (Patched1, smoothened or Gli1) has been associated with urothelial carcinoma initiation and progression [23–25] and in regulating cancer stem cells activities [26]. It has been reported that Shh-expressing basal cells give rise to muscle-invasive bladder cancer phenotype [27]. Interestingly, targeted inhibition of sonic hedgehog or one of its signaling components has recently been recognized to be one of the key targets that would have significant clinical implications in novel cancer therapeutics [28, 29]. The aim of the current study was to investigate the expression of sonic hedgehog protein in patients with bladder cancer by immunohistochemistry. Correlation analysis between Shh expression and patients’ clinico-pathological parameters and its prognostic value as a biomarker in bladder cancer was also evaluated.

## Materials and Methods

### Patients and Samples Collection

Tissue specimens were collected from bladder cancer patients who underwent surgical resections at King Abdulaziz University hospital between 2005 and 2011. A cohort samples from 128 patients were formalin fixed and embedded in paraffin and stored, in the department of pathology within the same institution, until use. This study was ethically approved by the institutional research ethics committee, faculty of medicine, King Abdulaziz University (ref. N. 149–14).

### Data Collection

Demographical, clinical and pathological data related to the collected tissue samples were also gathered and maintained in database. The collected information includes patients’ age, gender, marital status, tumour grade, stage, smoking. The mean follow-up was 30 months (range from 1 to 138 months). None of the patients had received chemo/radiation therapy before the surgery.

### Tissue Microarray (TMA) Construction

Formalin-fixed paraffin-embedded blocks were used for tissue microarray construction. Two cores (0.6 mm each) from each patient were integrated into a recipient block using a tissue chip microarrayer. A total of 128 blocks of bladder cancer were successfully transferred to construct four TMA slides making the total of 256 tissue cores in the TMA (Table 1). Hematoxylin & Eosin staining was done to obtain representative tumour cores and confirm the presence of tumour.

**Table 1:** Clinicopathological characteristics of the urothelial bladder cancer cases included in the study

Parameters			Bladder cancer (%)	
Sex	Male		108/131	82.443%
	Female		023/131	17.557%
Grade	High Grade		065/131	49.618%
	Low Grade		052/131	39.695%
	Unknown		014/131	10.687%
Age	< 60 Years		053/131	40.458%
	≥ 60 Years		076/131	58.015%
	Unknown		002/131	01.527%
Blood Group	A^+^		022/131	16.794%
	A^−^		001/131	00.763%
	B^+^		019/131	14.504%
	AB^+^		003/131	02.290%
	O^+^		038/131	29.008%
	O^−^		003/131	02.290%
	Unknown		045/131	34.351%
Type of Cancer	MIBC		058/131	44.275%
	NMIBC		049/131	37.405%
	Undecided		024/131	18.321%
Subtypes	Transitional		122/131	93.12%
	Squamous		006/131	04.580%
	Adenocarcinoma		001/131	00.763%
	Unknown		003/131	02.290%
Smoking	YES		041/131	31.298%
	NO		026/131	19.847%
	Unknown		064/131	48.855%
Survival	Died of Disease		038/131	29.008%
	Alive		091/131	69.466%
	Unknown		002/131	01.527%
Recurrence			051/131	38.931%

### Immunohistochemistry Staining

Immunohistochemical staining was performed using the Bench-Mark XT automated staining system (Ventana Medical Systems, Inc., Tucson, AZ, USA) according to the manufacturers’ instructions and as previously described [27]. Briefly, slides were de-waxed using EZprep solution for 30 min at 75 °C. Antigen retrieval was achieved using CC1 cell conditioning buffer for 64 min at 95 °C, then the slides were incubated with the primary rabbit anti-human sonic hedgehog polyclonal antibody (Spring Bioscience, USA) at a dilution of 1:100 for 30 min. Slides were then incubated with DAB chromogen detection kit as follow: application of universal DAB Inhibitor, then, universal DAB chromogen, then universal DAB H_2_O_2_, and last DAB Copper. Counterstaining was performed with hematoxylin and bluing reagent for 4 min. The sections were then dehydrated with ethanol then xylene and permanently mounted with mounting media and coverslip.

### Scoring and Data Analysis

Scoring of the IHC staining was performed using light microscope (X40 objective). Sub-cellular localization and the intensity of the staining were recorded during the scoring process. For the intensity of the cytoplasmic staining, four categories were used on scale 0 to 3: 0 (−) = negative: no detectable staining; 1(+) = weak, but still detectable staining; 2 (++) = moderate, clearly positive but still weak: and 3 (+++) = strong staining. To calculate the staining index both the intensity of staining and the proportion of positively-stained cells were taken into consideration, using the following formula as previously reported [27]:

(Ι = 0*f0 + 1*f1 + 2*f2 + 3*f3), where I is the staining index, f0-f3 are the proportions of the cells showing a defined level of staining intensity (from 0 to 3). The staining index ranges from 0 to 300. For association analysis between the level of SHh expression and patients’ clinical and pathological parameters Fisher’s two-sided exact tests was applied. Correlation between SHh levels and cancer-specific survival, Kaplan–Meier curve was used and *p* value was calculated using the log-rank test.

### Statistical Analysis

Statistical analysis was performed using SPSS (version 21). Fisher’s tests were used to analyse the association between sonic hedgehog expression and clinicopathological features and results were considered significant for values of *p* < 0.05. Kaplan–Meier curve was used to evaluate the effect of Shh expression on overall patients’ survival; *p* value was calculated using the log rank test.

## Results

### Patient Characteristics

Our cohort subjects included 128 bladder cancer patients, 105 males (82%) and 23 females (18%). The age of our cohort ranged from 26 to 93 years with a mean value of 61 years. Tumour metastasis was detected in 14 patients and recurrence was observed in 35% of the tested group (Table 1).

### Sonic Hedgehog Expression in Bladder Cancer

**Fig. 1:**
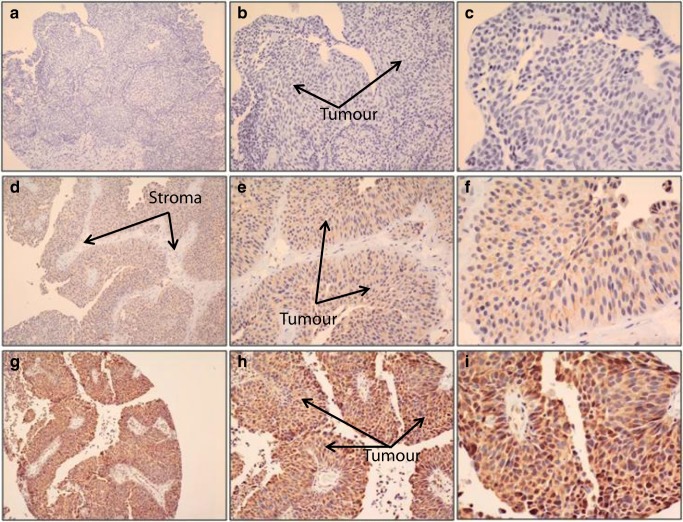
Expression of sonic hedgehog (SHh) in bladder cancer. Immunohistochemical staining of bladder cancer tissue microarray using Shh antibody. **a**, **b** and **c**. No Shh expression. **d**, **e** and **f**. Moderate Shh expression. **g**, **h** and **i**. Strong Shh expression. Images were taken using different objectives (×10, ×20, ×40)

In order to analyze the expression pattern of sonic hedgehog protein in bladder cancer, immunohistochemical staining of tissue microarrays, containing core biopsies from 128 patients affected with bladder cancer, was performed using hedgehog-targeted antibody. Assessment of the staining pattern revealed that sonic hedgehog protein was predominantly localized in the cytoplasm of the bladder cancer cells, as illustrated in Fig. 1. Variation in intensity of sonic hedgehog expression in the bladder cancer specimens was scored as follows: 0 (*n* = 33), 1 (*n* = 80), 2 (*n* = 15), 3 (*n* = 0). The receiver operating characteristic (*ROC) curve was used to* determine the cutoff that can be used to discriminate between high and low sonic hedgehog expression. Forty nine percent (49%) of the analyzed cohort exhibited high cytoplasmic expression of sonic hedgehog. The intensity and distribution of nuclear staining was not reported as significant while membranous localization of Shh was not seen (Fig. 1).

### Association between Sonic Hedgehog Expression and Clinicopathological Parameters

**Fig. 2:**
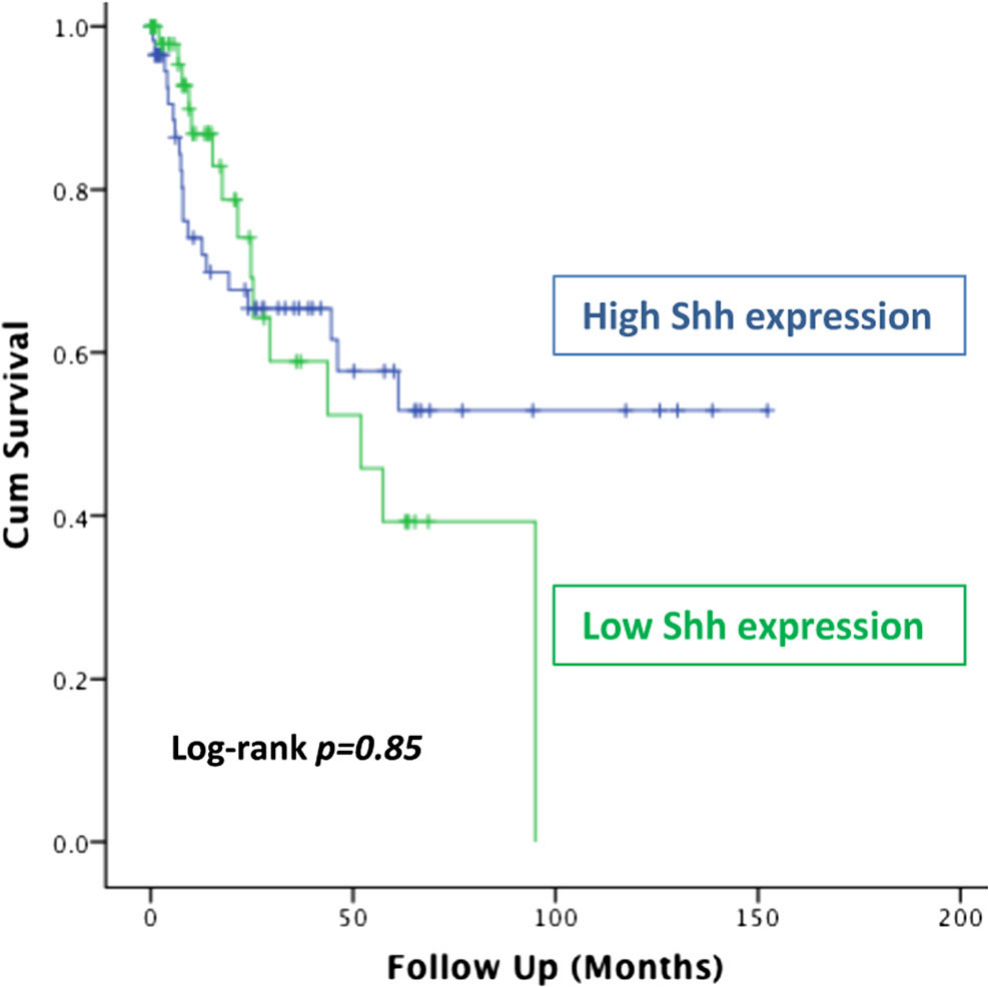
Overall survival of patients with bladder cancer. Kaplan-Meier curve showing no survival difference based on sonic hedgehog expression (*log-rank p = 0.85*)

All 128 patients were included in the analysis. Correlation analysis of Shh staining was undertaken to examine the relationship between the protein levels of Shh and any of the patients’ clinicopathological features. Our data indicated that the expression of Shh is significantly associated with lymph node invasion in bladder cancer patients (*p* = *0.02*). Statistical analysis indicated no correlation between the expression of Shh and other clinical parameters such as tumor grade, stage, smoking status or gender (Table 2). Similarly, there was no significant difference between negative and positive sonic hedgehog expression status and patients’ overall survival (Fig. 2).

**Table 2:** Association between sonic hedgehog protein expression and clinicopathological parameters in urothelial bladder cancer

Clinicopathological parameters	Number	Sonic hedgehog expression		*p* value
		Low (%)	High (%)	
Age group (years)	127			*ns*
< 60		23 (45.1%)	28 (54.9%)	
≥ 60		41 (53.9%)	35 (46.1%)	
Marital status	113			*ns*
Single		3 (50.0%)	3 (50.0%)	
Married		55 (51.4%)	52 (48.6%)	
Gender	128			*ns*
Male		51 (48.6%)	54 (51.4%)	
Female		14 (60.9%)	9 (39.1%)	
Smoking	65			*ns*
Yes		23 (56.1%)	18 (43.9%)	
No		14 (58.3%)	10 (41.7%)	
Type of cancer	110			*ns*
MIBC		27 (46.6%)	31 (53.4%)	
NMIBC		28 (53.8%)	24 (46.2%)	
Histological grade	113			*ns*
Low grade		23 (46.0%)	27 (54.0%)	
High grade		34 (54.0%)	29 (46.0%)	
Family history	65			*ns*
Yes		6 (85.7%)	1 (14.3%)	
No		30 (51.7%)	28 (48.3%)	
Lymph node status	83			***0.02***
Negative		43 (59.7%)	29 (40.3%)	
Positive		2 (18.2%)	9 (81.8%)	
Metastasis	80			*ns*
Positive		6 (42.9%)	8 (75.1%)	
Negative		40 (60.6%)	26 (39.4%)	
Recurrence	127			*ns*
Yes		23 (52.3%)	21 (47.7%)	
No		41(49.4%)	42 (50.6%)	

*ns* not significant

## Discussion

Sonic hedgehog is a member of hedgehog family of small secreted proteins, that were originally discovered as important regulator during vertebrates development [28]. It is well documented that Shh is expressed in normal bladder epithelium to maintain the regenerative potential of the epithelium and this expression exhibited different spatial and temporal distribution during abnormal bladder development indicating the important role of Shh in bladder tumorigenesis [30, 31]. Recent findings revealed that deregulation of sonic hedgehog pathway is associated with plethora of malignancies in various tissue-types through mutations in Patched (Ptch1) and/or the G protein-coupled receptor smoothened (SMO) genes [29, 32]. The potential oncogenic role of sonic hedgehog and the components of its signaling pathway on bladder pathogenesis is not well delineated. However several attempts have been made and reports indicated the involvement of Shh in bladder cancer growth and tumorigenicity [22, 33, 34]. Chen et al. (2010) undertook genotyping analysis on 177 single-nucleotide polymorphisms (SNP) using 803 bladder cancer cases and equal number of healthy controls and found that germ-line genetic variations in the Shh pathway predicted clinical outcomes of non-muscle-invasive bladder cancer patients receiving transurethral resection and BCG treatment [35]. In an independent study, Shin et al. 2011 demonstrated increased levels of Shh and Gli1 mRNAs in response to bladder tissue injury suggesting that Shh increase the proliferative potential of bladder stem cells. The latter deemed responsible for bladder cancer recurrence and drug resistance [36].

Our study showed high expression of Shh protein in the cytoplasmic compartment of bladder cancer using immunohistochemistry. The high expression was seen in almost 50% of the tested cohort. Interestingly, the expression of Shh protein significantly correlated with lymph node metastasis of tumours. This was consistent with previous findings by He et al. (2012) and Pignot et al. (2012), both  authors  reported that the levels of Shh, Ptch1 and Gli1 proteins were significantly elevated in bladder cancer tissues. Over-expression of these proteins correlated with a number of pathological parameters including lymph node metastasis [37, 38]. In an attempt to decipher the mechanism by which activation of Shh pathway mediate the tumorigenic potential and malignant phenotype in bladder cancer, Islam et al. (2016) performed a number of in-vitro and in-vivo experiments and revealed that SHh was able to induce the epithelial-to-mesenchymal phenotype (EMT) through activation of the transforming growth factor beta (TGF-b1) pathway [26]. It has recently been found that Shh plays an important role in urothelial bladder cancer by promoting bladder cancer stem cells self-renewal [39–41] which may also explain the elevated rates of tumour recurrence and resistance to conventional chemo/radiotherapies associated with bladder cancer. Involvement of sonic hedgehog in regulating bladder cancer stem cells activities could also add to the burden of the aggressive phenotype of bladder cancer, thus, targeting Shh pathway with curcumin or another chemopreventive agent might be an effective strategy for inhibiting bladder cancer development [42].

In conclusion, there is clear evidence that Shh signaling pathway is abnormally activated in several cancers including urothelial carcinoma of the bladder and the underlying mechanism is yet to be determined. This pathway represents a target for potential anti-cancer therapy and controlling recurrence in urothelial bladder carcinoma.

## References

[1] Antoni S, Ferlay J, Soerjomataram I, Znaor A, Jemal A, Bray F (2017). Bladder Cancer incidence and mortality: a global overview and recent trends. Eur Urol.

[2] Sanli O, Dobruch J, Knowles MA, Burger M, Alemozaffar M, Nielsen ME, Lotan Y (2017). Bladder cancer. Nat Rev Dis Primers.

[3] Morgan TM, Clark PE (2010). Bladder cancer. Curr Opin Oncol.

[4] Kaufman DS, Shipley WU, Feldman AS (2009). Bladder cancer. Lancet.

[5] Millán-Rodríguez F, Chéchile-Toniolo G, Salvador-Bayarri J, Palou J, Algaba F, Vicente-Rodríguez J (2000). Primary superficial bladder cancer risk groups according to progression, mortality and recurrence. J Urol.

[6] Cheung G, Sahai A, Billia M, Dasgupta P, Khan MS (2013). Recent advances in the diagnosis and treatment of bladder cancer. BMC Med.

[7] Netto GJ, Tafe LJ (2016). Emerging bladder Cancer biomarkers and targets of therapy. Urol Clin North Am.

[8] Cheng L, Davison DD, Adams J, Lopez-Beltran A, Wang L, Montironi R, Zhang S (2014). Biomarkers in bladder cancer: translational and clinical implications. Crit Rev Oncol Hematol.

[9] Mbeutcha A, Lucca I, Mathieu R, Lotan Y, Shariat SF (2016). Current status of urinary biomarkers for detection and surveillance of bladder Cancer. Urol Clin North Am.

[10] Ehlen HW, Buelens LA, Vortkamp A (2006). Hedgehog signaling in skeletal development. Birth Defects Res C Embryo Today.

[11] Song J, Zhang J, Wang J, Guo X, Dong W (2015). β1 integrin mediates colorectal cancer cell proliferation and migration through regulation of the hedgehog pathway. Tumour Biol.

[12] Kanaya K, Ii M, Okazaki T (2015). Sonic hedgehog signaling regulates vascular differentiation and function in human CD34 positive cells: vasculogenic CD34(+) cells with sonic hedgehog. Stem Cell Res.

[13] Velcheti V, Govindan R (2007). Hedgehog signaling pathway and lung cancer. J Thorac Oncol.

[14] Statkiewicz M, Maryan N, Lipiec A, Grecka E, Grygorowicz MA, Omiotek M, Gorska A, Mikula M, Malecki M (2014). The role of the SHH gene in prostate cancer cell resistance to paclitaxel. Prostate.

[15] Tao Y, Mao J, Zhang Q, Li L (2011). Overexpression of hedgehog signaling molecules and its involvement in triple-negative breast cancer. Oncol Lett.

[16] Yoshimoto AN, Bernardazzi C, Carneiro AJ (2012). Hedgehog pathway signaling regulates human colon carcinoma HT-29 epithelial cell line apoptosis and cytokine secretion. PLoS One.

[17] Liao X, Siu MK, Au CW (2009). Aberrant activation of hedgehog signaling pathway in ovarian cancers: effect on prognosis, cell invasion and differentiation. Carcinogenesis.

[18] Maréchal R, Bachet JB, Calomme A (2015). Sonic hedgehog and Gli1 expression predict outcome in resected pancreatic adenocarcinoma. Clin Cancer Res.

[19] Giakoustidis A, Giakoustidis D, Mudan S, Sklavos A, Williams R (2015). Molecular signalling in hepatocellular carcinoma: role of and crosstalk among WNT/ß-catenin, sonic hedgehog, notch and Dickkopf-1. Can J Gastroenterol Hepatol.

[20] Mullor JL, Dahmane N, Sun T, Ruiz i Altaba A (2001). Wnt signals are targets and mediators of Gli function. Curr Biol.

[21] Bosco-Clément G, Zhang F, Chen Z, Zhou HM, Li H, Mikami I, Hirata T, Yagui-Beltran A, Lui N, Do HT, Cheng T, Tseng HH, Choi H, Fang LT, Kim IJ, Yue D, Wang C, Zheng Q, Fujii N, Mann M, Jablons DM, He B (2014). Targeting Gli transcription activation by small molecule suppresses tumor growth. Oncogene.

[22] Shevde LA, Samant RS (2014). Nonclassical hedgehog-GLI signaling and its clinical implications. Int J Cancer.

[23] Thievessen I, Wolter M, Prior A, Seifert HH, Schulz WA (2005). Hedgehog signaling in normal urothelial cells and in urothelial carcinoma cell lines. J Cell Physiol.

[24] Fei DL, Sanchez-Mejias A, Wang Z, Flaveny C, Long J, Singh S, Rodriguez-Blanco J, Tokhunts R, Giambelli C, Briegel KJ, Schulz WA, Gandolfi AJ, Karagas M, Zimmers TA, Jorda M, Bejarano P, Capobianco AJ, Robbins DJ (2012). Hedgehog signaling regulates bladder cancer growth and tumorigenicity. Cancer Res.

[25] Sverrisson EF, Zens MS, Fei DL (2014). Clinicopathological correlates of Gli1 expression in a population-based cohort of patients with newly diagnosed bladder cancer. Urol Oncol.

[26] Islam SS, Mokhtari RB, Noman AS, Uddin M, Rahman MZ, Azadi MA, Zlotta A, van der Kwast T, Yeger H, Farhat WA (2016). Sonic hedgehog (Shh) signaling promotes tumorigenicity and stemness via activation of epithelial-to-mesenchymal transition (EMT) in bladder cancer. Mol Carcinog.

[27] Shin K, Lim A, Odegaard JI, Honeycutt JD, Kawano S, Hsieh MH, Beachy PA (2014). Cellular origin of bladder neoplasia and tissue dynamics of its progression to invasive carcinoma. Nat Cell Biol.

[28] Kelleher FC (2011). Hedgehog signaling and therapeutics in pancreatic cancer. Carcinogenesis.

[29] Di Magno L, Coni S, Di Marcotullio L, Canettieri G (2015). Digging a hole under hedgehog: downstream inhibition as an emerging anticancer strategy. Biochim Biophys Acta.

[30] Chen M, Hildebrandt MA, Clague J (2010). Genetic variations in the sonic hedgehog pathway affect clinical outcomes in non-muscle-invasive bladder cancer. Cancer Prev Res (Phila).

[31] Shin K, Lee J, Guo N, Kim J, Lim A, Qu L, Mysorekar IU, Beachy PA (2011). Hedgehog/Wnt feedback supports regenerative proliferation of epithelial stem cells in bladder. Nature.

[32] Buhmeida A, Dallol A, Merdad A, al-Maghrabi J, Gari MA, Abu-Elmagd MM, Chaudhary AG, Abuzenadah AM, Nedjadi T, Ermiah E, al-Thubaity F, al-Qahtani MH (2014). High fibroblast growth factor 19 (FGF19) expression predicts worse prognosis in invasive ductal carcinoma of breast. Tumour Biol.

[33] Ingham PW, McMahon AP (2001). Hedgehog signaling in animal development: paradigms and principles. Genes Dev.

[34] DeSouza KR, Saha M, Carpenter AR, Scott M, McHugh KM (2013). Analysis of the sonic hedgehog signaling pathway in normal and abnormal bladder development. PLoS One.

[35] Gupta S, Takebe N, Lorusso P (2010). Targeting the hedgehog pathway in cancer. Ther Adv Med Oncol.

[36] Aboulkassim TO, LaRue H, Lemieux P, Rousseau F, Fradet Y (2003). Alteration of the PATCHED locus in superficial bladder cancer. Oncogene.

[37] Pignot G, Vieillefond A, Vacher S, Zerbib M, Debre B, Lidereau R, Amsellem-Ouazana D, Bieche I (2012). Hedgehog pathway activation in human transitional cell carcinoma of the bladder. Br J Cancer.

[38] He HC, Chen JH, Chen XB, Qin GQ, Cai C, Liang YX, Han ZD, Dai QS, Chen YR, Zeng GH, Zhu JG, Jiang FN, Zhong WD (2012). Expression of hedgehog pathway components is associated with bladder cancer progression and clinical outcome. Pathol Oncol Res.

[39] van der Horst G, Bos L, van der Pluijm G (2012). Epithelial plasticity, cancer stem cells, and the tumor-supportive stroma in bladder carcinoma. Mol CancerRes.

[40] Li C, Du Y, Yang Z, et al. (2015) GALNT1-mediated glycosylation and activation of sonic hedgehog signaling maintains the self-renewal and tumor-initiating capacity of bladder Cancer stem cells. Cancer Res 10.1158/0008-5472.CAN-15-2309 26676748

[41] Syed IS, Pedram A, Farhat WA (2016). Role of sonic hedgehog (Shh) signaling in bladder Cancer Stemness and tumorigenesis. Curr Urol Rep.

[42] Wang D, Kong X, Li Y, Qian W, Ma J, Wang D, Yu D, Zhong C (2017). Curcumin inhibits bladder cancer stem cells by suppressing sonic hedgehog pathway. Biochem Biophys Res Commun.

